# Rapid analytical methods for the microalgal and cyanobacterial biorefinery: Application on strains of industrial importance

**DOI:** 10.1002/mbo3.1156

**Published:** 2021-02-28

**Authors:** Joseph S. Palmer, Linda A. Lawton, Rocky Kindt, Christine Edwards

**Affiliations:** ^1^ School of Pharmacy and Life Sciences Robert Gordon University Aberdeen UK; ^2^ ScotBio BioCity Scotland Newhouse UK

**Keywords:** biochemical analysis, carotenoids, liquid chromatography, mass spectrometry, phycobiliproteins

## Abstract

To realize the potential of microalgae in the biorefinery context, exploitation of multiple products is necessary for profitability and bioproduct valorization. Appropriate analytical tools are required for growth optimization, culture monitoring, and quality control purposes, with safe, low‐tech, and low‐cost solutions favorable. Rapid, high‐throughput, and user‐friendly methodologies were devised for (a) determination of phycobiliproteins, chlorophylls, carotenoids, proteins, carbohydrates, and lipids and (b) qualitative and quantitative carotenoid profiling using UPLC‐PDA‐MS^E^. The complementary methods were applied on 11 commercially important microalgal strains including prasinophytes, haptophytes, and cyanobacteria, highlighting the suitability of some strains for coproduct exploitation and the method utility for research and industrial biotechnology applications. The UPLC method allowed separation of 41 different carotenoid compounds in <15 min. Simple techniques are described for further quantification and comparison of pigment profiles, allowing for easy strain selection and optimization for pigment production, with suitability for biotechnological or biomedical applications.

## Graphical abstract

Rapid, user‐friendly methodologies were devised for the determination of phycobiliprotein, pigment, and macromolecule contents in microalgae, and for qualitative and quantitative carotenoid profiling using liquid chromatography coupled with spectrophotometry and mass spectrometry. Methods were applied to commercially important microalgal strains, demonstrating their application and suitability as tools for quality control, strain selection, and growth optimization purposes within microalgal research and integrated biorefinery settings.

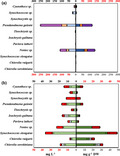

## 1 INTRODUCTION

The microalgal biorefinery concept is an increasingly attractive strategy for the clean and sustainable production of foods, fuels, pharmaceuticals, and chemicals needed for a growing population faced with various environmental challenges. Changing consumer preferences for healthier, more environmentally friendly products is driving industrial production away from petrochemicals and toward naturally synthesized compounds (Adarme‐Vega et al., 2012). By utilizing only light, nutrients, and carbon dioxide, microalgae represent a highly efficient means of producing high‐quantity and high‐quality products with minimal space and resource demands (Gilbert‐López et al., 2015). Key microalgal‐derived products include whole biomass, protein, polyunsaturated fatty acids, and pigments, plus emerging bioactive compounds such as fucoxanthin, sulfated polysaccharides, phytosterols, and alkaloids (Jacob‐Lopes et al., 2019). Single product production strategies from microalgae have rarely proved economically successful, with barriers such as low biomass concentrations and high downstream processing costs proving prohibitive (Zhu, 2015). Microalgal industries must therefore focus on a multiple‐product biorefinery approach, with growth strategies requiring a more holistic approach of co‐optimizing different operations, rather than hyperaccumulation of a single product (Chaiklahan et al., 2018; Gifuni et al., 2019). Leftover biomass is valorized by a recovery of coproducts, thus improving revenues and reducing waste. This allows a cleaner and greener approach to manufacturing, particularly when green downstream processing techniques are adopted, allowing for integration into the circular bioeconomy (Gifuni et al., 2019; Marzorati et al., 2020).

Appropriate strain selection and optimization of growth conditions represent key steps for microalgal biorefinery development and upstream processing; however, the need for appropriate tools for detecting, quantifying, and monitoring target compounds must not be overlooked. Suitable methods are not only essential for profiling microalgal strains for the production of high‐value or bioactive compounds but also fundamental for research, quality control, and culture monitoring purposes. While such tools must offer an appropriate level of sensitivity and robustness, they also need to be high‐throughput, and cost‐, time‐, and sample‐efficient for successful utilization in industrial settings. Although various methods exist in the literature for the separate profiling and quantification of carotenoids, phycobiliproteins, and macronutrients, little consideration is given to other useful requirements such as their rapidity and simplicity, utilization of low‐cost, green, safer reagents, suitability for use on various microalgal strains, and whether different assays are compatible within a unified methodology (Chen & Vaidyanathan, 2013).

Considering the requirements above, a methodology was devised that encompasses various sensitive, high‐throughput, and easy‐to‐use assays into one unified process for analysis of various intracellular pigments and key macromolecule classes. The focus was placed on the use of non‐toxic, affordable reagents, and basic laboratory instruments. A second, complementary method is also described for the rapid qualitative and quantitative untargeted analysis of carotenoids. This assay allows a quick, sensitive, and high‐resolution identification and quantification of major carotenoids and their common isomers, allowing a comparison of carotenoid profiles between strains. While more intensive methods may be more applicable for the determination of specific components, such as the Kjeldahl method for total protein determination, the methods described in this work have broad application within biotechnology and as routine methods for food analysis, chemotaxonomy, and biomedical purposes. Low sample requirements, rapid processing, and low‐tech assays facilitate its use for many applications. The full protocol is demonstrated on 11 non‐toxic, commercially important microalgal species that are documented as potential candidates for commercial exploitation. This includes haptophytes, microalgae capable of significant bioactive accumulation (Gilbert‐López et al., 2015), Prasinophytes, fast‐growing green alga commonly exploited by microalgal industries (’t Lam et al., 2017), and cyanobacteria, ancient photoautotrophs that produce high‐value carotenoids and phycobiliproteins (Park et al., 2018).

## 2 MATERIALS AND METHODS

### 2.1 Chemicals and reagents

All American Chemical Society (ACS) grade anthrone, copper sulfate pentahydrate, phosphoric acid (85%), sodium hydroxide, sulfuric acid and vanillin, butylated hydroxytoluene, bovine serum albumin, starch, and LC‐MS grade acetone, ethanol, and isopropanol were purchased from Sigma‐Aldrich. LC‐MS grade acetonitrile, methanol, and formic acid were purchased from Fisher Scientific, Loughborough UK. Canola oil was obtained from a local supermarket. Deionized water (DW) was provided by a Milli‐Q system (Millipore), while authentic β‐carotene, β‐cryptoxanthin, echinenone, zeaxanthin, and mixed phytoplankton standards were obtained from DHI lab products, Denmark.

### 2.2 Microalgae and culture conditions

*Cyanothece* sp. PCC 7425, *Synechococcus* sp. PCC 7002/2, *Synechocystis* sp. PCC 6803 (Pasteur Culture Collection of Cyanobacteria, Paris, France), *Pseudanabaena galeate* CCNP 1313, *Nostoc* sp. CCNP 1411 (Culture Collection of Northern Poland, Gdansk, Poland), *Synechococcus elongatus* UTEX 2973 (UTEX Culture Collection of Algae, Austin, TX, USA), *Chlorella sorokiniana*, and *C*. *vulgaris* were grown on DW enriched with BG‐11 medium (100 ml medium in 250 ml Erlenmeyer flasks; Stanier et al., 1971). *Isochrysis galbana*, *Pavlova lutheri*, and *Tisochrysis* sp., obtained from CEFAS (Centre for Environment, Fisheries, and Aquaculture Science), were grown on seawater enriched with F2‐Si medium (100 ml medium in 250‐ml conical flasks; Guillard & Ryther, 1962). All cultures were grown at 22 ± 1°C with 12:12‐h light and dark cycles at 20 µmol m^−2 ^s^−1^ for 35 days. To harvest, 1.5 ml aliquots of culture were centrifuged in Eppendorf tubes (1.5 ml) at 4,000 *g* for 10 min. The supernatant was discarded, and the pellets were lyophilized. Pellets were weighed and stored at −20ºC until analysis.

### 2.3 A unified method for total pigment and macromolecule determination

The method described here is the optimized procedure for the determination of 10 key components, all utilizing simple colorimetric methods. For all spectrophotometric readings, an additional reading at 800 nm was taken for baseline correction between spectra. All color‐developing reagents (biuret, phospho‐vanillin [PV], and anthrone) should be freshly made immediately before analysis. Four replicates were performed for each strain.

#### 2.3.1 Phycobiliprotein determination

Lyophilized cell pellets were extracted in 1 ml of DW, vortexed for 30 s, and centrifuged at 4,000 *g* for 10 min. The supernatant was carefully poured into a microcuvette and co‐read at 562, 615, and 652 nm, after blanking the spectrophotometer with DW. The concentrations of C‐phycocyanin (CPC), allophycocyanin (APC), and phycoerythrin (PE) (mg ml^−1^) were calculated using the formulae of Bennett and Bogobad (1973):

$$\text{CPC}\;={\;A}_{615}‐\;0.474\left(A_{652}\right)/5.34$$

$$\text{APC}\;={\;A}_{652}‐\;0.208\left(A_{615}\right)/5.09$$

$$\text{PE}\;={\;A}_{562}‐2.41\left(\text{CPC}\right)\;‐\;0.849\left(\text{APC}\right)/9.62$$

#### 2.3.2 Total carotenoid and pigment determination

To the remaining pellet, 1 ml of acetone was added, vortexed for 30 s, and centrifuged at 4,000 *g* for 10 min. The supernatant was carefully transferred into a 2‐ml amber glass vial. The extraction was repeated with 1 ml of acetone, and the supernatants are pooled. An aliquot was read at 470, 645, 653, 654, and 662 nm after blanking with acetone. The total carotenoid (TC), chlorophyll a, (Ca) chlorophyll b (Cb), and total pheophytin (TC) concentrations (μg ml^−1^) were determined using the formulae of Hynstova et al. (2018):

$$\text{Ca}=11.24\left(A_{662}\right)\;‐2.04\left(A_{645}\right)$$

$$\text{Cb}=20.13\left(A_{645}\right)\;‐4.19\left(A_{662}\right)$$

$$\text{TC}\;=\;(1000\left(A_{470}\right)\;‐1.9\left(\text{Ca}\right)\;‐63.14\left(\text{Cb}\right))/214$$

$$\text{TP}=321.3\left(A_{653}\right)\;‐208.4\left(A_{654}\right)$$

After this point, the remaining pellet should be a pale, translucent color. The above assays can be repeated as necessary to remove any residual pigments.

#### 2.3.3 Macromolecule extraction

The cell pellet from the previous step was resuspended in 1 ml of lysis buffer (1 M NaOH in 25% MeOH (Chen & Vaidyanathan, 2013)) and incubated at 70ºC for 10 min. The tube was chilled on ice and 500 µl transferred to a 2‐ml screw‐top vial preloaded with 600 mg of 0.5 mm glass beads. This was disrupted by bead beating using a cell disruptor for 5 min (Genie Disruptor; Scientific Industries). The remaining sample solution was added to the vial and disrupted for another 5 min, before centrifuging at 13,700 *g* for 1 min. The lysate (supernatant) was used for protein, carbohydrate, and lipid analysis.

#### 2.3.4 Protein analysis

Soluble proteins were quantified using the microbiuret method (Itzhaki & Gill, 1964). For the biuret reagent, 20 ml of 1% aqueous CuSO_4_·5H_2_O (*w*/*v*) was added to 50 ml of 30% NaOH in a volumetric flask and topped up to 100 ml with DW. Cell lysate (400 µl) was then mixed with 400 µl of the reagent and vortexed briefly; then, the color formation was monitored at 310 nm after blanking the spectrophotometer with lysis buffer. Bovine serum albumin (BSA), dissolved in lysis buffer, was used to create calibration curves for protein quantification.

#### 2.3.5 Lipid analysis

Lipid analysis was performed using the sulfo‐phospho‐vanillin (SPV) assay, based on the method of Park et al., (2016). The PV reagent was made by dissolving 200 mg of vanillin powder in 8 ml of ethanol. This was mixed with 72 ml of DW before slowly adding 20 ml of 85% phosphoric acid. The mixture was stirred well and kept in the dark until use. Sulfuric acid (500 µl) was then added to 100 µl of cell extract in glass HPLC vials. Vials were capped, briefly vortexed, and incubated at 90°C for 10 min. The vial was then chilled on ice for 5 min, before adding 300 µl of PV reagent. This was vortexed, then incubated in the dark for 10 min. Samples were read at 530 nm after blanking the spectrophotometer with 75% sulfuric acid. To create the calibration curve, Canola oil was solubilized in acetone and diluted accordingly in acetone. Aliquots (100 µl) of each concentration were added to vials, the acetone was evaporated off, and then, 100 µl of lysis buffer was added to resuspend oil. Standards were then processed in the same manner as samples.

#### 2.3.6 Carbohydrate analysis

Total carbohydrate quantification was performed using the anthrone method described in Chen and Vaidyanathan, (2013). In glass vials, 150 µl of cell lysate was mixed and vortexed with 300 µl of cold sulfuric acid. To this, 600 µl of 0.2% anthrone in 75% sulfuric acid (*w*/*v*) was added, capped, vortexed, and incubated at 100°C for 15 min. Samples were read at 625 nm after blanking with 75% sulfuric acid. Starch was used as a standard for calibration.

### 2.4 Method for individual carotenoid identification and quantification

#### 2.4.1 Pigment extraction

Lyophilized cell pellets were resuspended in 1 ml of cold absolute ethanol containing 0.1% (*w*/*v*) butylated hydroxytoluene (BHT) and transferred into 2‐ml screw‐top polystyrene tubes with 0.5 g of 0.5 mm glass beads. Tubes were briefly vortexed and sonicated for 10 min in iced water using an ultrasonic bath (Decon Ultrasonics Ltd) before disrupting for 5 min by bead beating. The tubes were then centrifuged at 13,700 *g* for 10 min. The supernatant was transferred to clean centrifuge tubes. A further 500 µl ml of cold ethanol +0.1% BHT was added to the pellet with bead beating and centrifuging steps repeated as described, with the supernatant pooled with the previous extract.

#### 2.4.2 Saponification

The extract was then saponified to hydrolyze esterified carotenoids and to separate chlorophyll and chlorophyll derivatives from carotenoid compounds. Five hundred microliters of cold, 40% NaOH was added to 1 ml of extract in 10‐ml glass collection vials, and the samples were flushed with N_2_ gas for 3 min. Samples were then shaken at 1,000 rpm for 5 min. These steps were all performed in dim light.

#### 2.4.3 Carotenoid separation

Two milliliters of heptane: dichloromethane (4:1, *v*/*v*) +0.1% BHT were added to the saponified extracts and were shaken for 5 min at 2,000 rpm. One milliliter of DW was added, and the mixture was vortexed for 30 s, before centrifuging at 700 *g* for 5 min. The upper organic layer was carefully removed by pipette and aliquoted into amber glass vials (2 × 750 µl), which were then dried under N_2_ and stored at −80°C until UPLC analysis.

#### 2.4.4 *UPLC*‐*QTOF*‐*MS^E^*

Carotenoids were qualitatively and quantitatively identified using ultra‐high‐performance liquid chromatography coupled to quadrupole time‐of‐flight mass spectrometry (UPLC‐QTOF‐MS^E^). Dried extracts were resuspended to the original volume (750 µl) in elution buffer (acetonitrile:methanol [7:3, *v*/v] +0.1% BHT), and 5 µl was injected into an Acquity UPLC System equipped with a photodiode array detector (Acquity PDA), directly coupled to a quadrupole time‐of‐flight mass spectrometer (XEVO‐QTOF‐MS) in series (Waters). Liquid chromatography was performed with an Accucore™ C_30_ column (Thermo Scientific) based on the protocol of Sommella et al. (2018), using mobile phases A (0.1% formic acid in MS grade H_2_O) and B (0.1% (*v*/*v*) formic acid in acetonitrile/methanol/isopropanol (7:2:1 *v*/*v*). All flow rates were 0.4 ml min^−1^ unless otherwise stated. Initial conditions were 60% B, 60%–75% B (0–0.75 min), 75–85% B (0.75–3.25 min), 85–95% B (3.25–5 min), isocratic at 95% B (5–7 min), isocratic at 100% B (7.01–9 min), 95%–90% B (9.01–10 min, (at 0.5 ml min^−1^), 90–60% B (10–11 min), and then, the mobile phase and column were allowed to equilibrate back to initial conditions for 2 min. The column and autosampler were maintained at 40 and 10°C, respectively. A blank sample wash injection was carried out between samples to ensure no carryover between samples. Absorbance was monitored between 300 and 700 nm, scan time was 10 points s^−1^ with the PDA module.

The MS was performed with an electrospray ionization (ESI) source in positive‐ion mode with centroid data acquisition. The MS^E^ functionality was used to collect parent ion mass/charge (*m*/*z*) between 350 and 1,200 *m*/*z* in low‐energy mode (6 V), before ramping the energy between 20 and 40 V to cause ion fragmentation, with higher energy fragments scanned over the range of 100–1,200 *m*/*z*, with an acquisition rate of 0.25 s. The mass spectrometer was operated at a cone voltage of 25 V, a capillary voltage of 3 kV, cone gas flow, and desolvation gas flows of 40 and 1,000 L H^−1^, respectively, and source and desolvation temperatures of 150 and 500°C, respectively.

### 2.5 Data analysis

Data were analyzed in MassLynx 4.1 software (Waters). Compounds with main spectral peaks outside the range of 330–550 nm and <1,000 μAU were excluded from the analysis. Carotenoids were identified by comparison of retention times (rt), parent ion *m*/*z* (after adduct subtraction), and fragmentation patterns to authentic standards. Identification of additional peaks was carried out by comparison of in‐source and MS^E^ fragmentation patterns and spectral data with published values. Quantification of β‐carotene, β‐cryptoxanthin, echinenone, and zeaxanthin was performed by comparison of spectral peak areas with corresponding calibration curves prepared from authentic standards. Lutein was quantified by comparison of peak areas with the zeaxanthin calibration curve. Quantification of other identified carotenoids was performed using the method described in Takaichi et al. (2009), whereby the molar extinction coefficient in the UPLC eluent at the maximum wavelengths of each carotenoid was assumed to be 140 mM^−1 ^cm^−1^. A data matrix of peak areas for detected carotenoids was exported to MetaboAnalyst (Chong et al., 2019) for partial least squares discriminant analysis (PLS‐DA). Quantile normalization and autoscaling options were used.

## 3 RESULTS AND DISCUSSION

### 3.1 Total pigment and macromolecule determination

**FIGURE 1:**
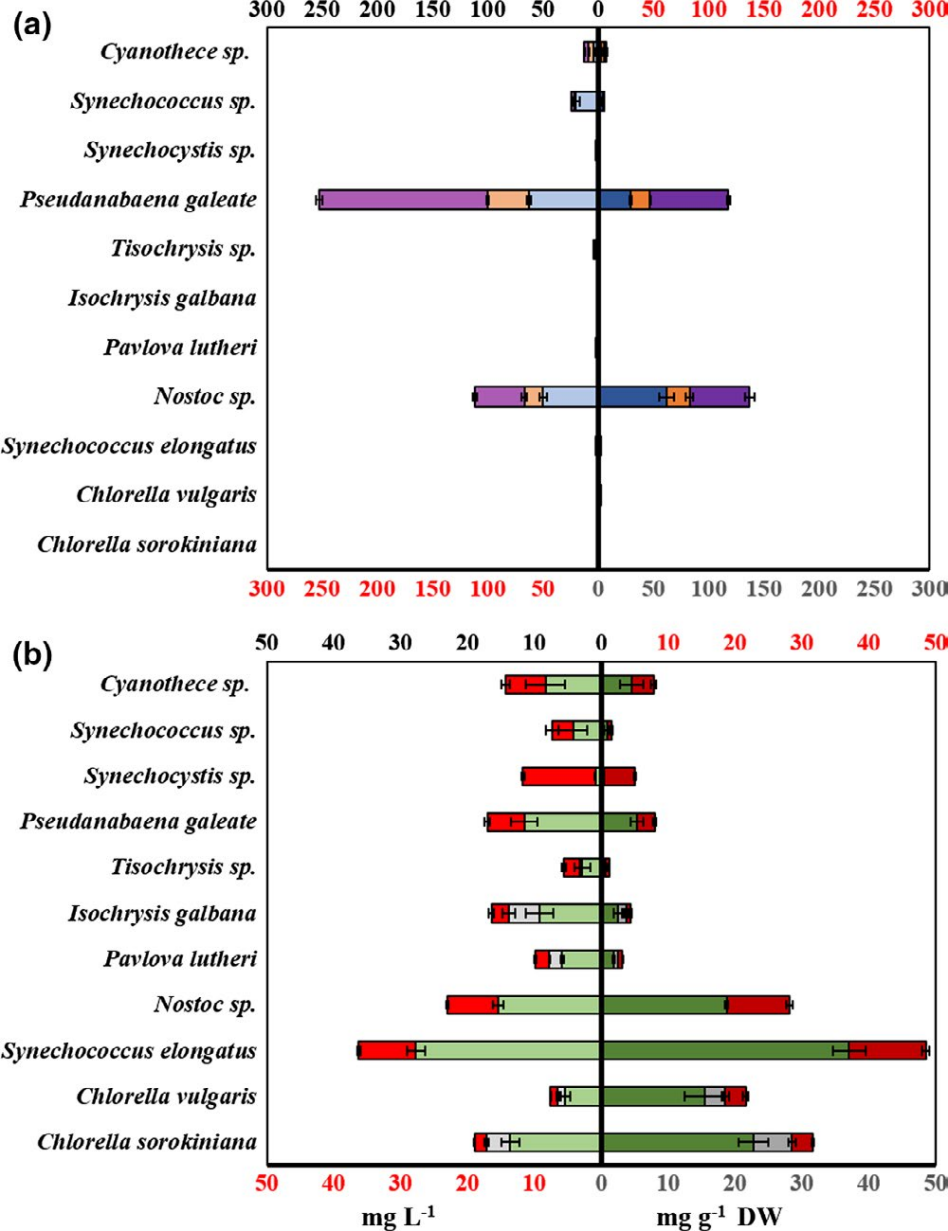
The concentration of pigments in algal biomass. Phycobiliproteins (a) C‐phycocyanin (blue), allophycocyanin (orange), and phycoerythrin (purple) and other pigments (b) chlorophyll a (green), chlorophyll b (gray), and total carotenoids (red) are shown in both mg L^−1^ culture medium and mg g^−1^ DW of algal biomass. (Bars show *SE* [*n* = 4])

In the first part of the unified methodology, two simple assays were used to determine concentrations of phycobiliproteins, total carotenoids, chlorophyll a, b, and pheophytin from the lyophilized pellets of 11 microalgal strains. Snap freezing or repeated freeze–thaw cycles can be used instead of lyophilization, in which case dry weight content can be determined independently. Phycobiliproteins were detected at significant amounts in two strains, *Nostoc* sp. CCNP 1411/1 and *Pseudanabaena galeate* CCNP 1313 comprising approximately 14% and 12% of the total dry weight, respectively (Figure 1a). In *Nostoc* sp. CCNP 1411/1, C‐phycocyanin (CPC) was the main phycobiliprotein (61.6 mg g^−1^), whereas phycoerythrin (PE) dominated in *P*. *galeate* CCNP 1313 (71.1 mg g^−1^). This is in close agreement with the concentrations and CPC:PE ratios reported in *P*. *galeate* CCNP 1313 by Cegłowska et al. (2020). Low concentrations of phycobiliproteins were also detected in *Cyanothece* sp. PCC 7425 and *Synechococcus* sp. PCC 7002/2 (less than 1% DW). *Synechococcus sp*. PCC 7002 does not naturally produce PE (Alvey et al., 2011), which was also observed in this work. The absence of phycobiliproteins in the other cyanobacterial strains may be due to low lighting (Hong & Lee, 2008) or degradation of phycobiliproteins caused by nutrient deprivation (Collier & Grossman, 1994). Chlorophyll a was present in all strains, with concentrations of over 15 mg g^−1^ in *Synechococcus elongatus* UTEX 2973, *Nostoc* sp. CCNP 1411/1, and both *Chlorella* strains (Figure 1b). Chlorophyll b was only detected in *Chlorella* strains, *Isochrysis galbana*, and *Pavlova lutheri*, albeit at low concentrations (<1.5 mg g^−1^) in the latter two strains. This corresponds well to the pigment compositions of an Indian *C*. *vulgaris* strain grown under a range of lighting conditions, as detailed in Sharma (2012). *S*. *elongatus* UTEX 2973 and *Nostoc* sp. CCNP 1313 had the highest total carotenoid concentrations, at 11.45 and 9.37 mg g^−1^, respectively (Figure 1b), while pheophytin concentrations were below <0.5 mg g^−1^ for all strains except *C*. *sorokiniana*, which contained 1.86 mg g^−1.^ Pheophytin is a degradation product of chlorophyll, and its determination can be used to indicate harvesting and post‐harvest processes, which may cause chlorophyll degradation (Hynstova et al., 2018). As pigments are sensitive to degradation by light and heat, the detailed protocol allows rapid determination of these components before the more vigorous mechanical and thermal lysis steps. A centrifugation protocol of 4,000 *g* for 10 min was selected for effective pelleting of cell material while allowing easy resuspension in subsequent steps. Higher centrifugation speeds resulted in difficulties in resuspending pellets and subsequent incomplete extraction of pigments. It was possible to determine the concentrations of seven different pigment components in under 30 minutes from lyophilized algal pellets, demonstrating the rapidity of the method.

**FIGURE 2:**
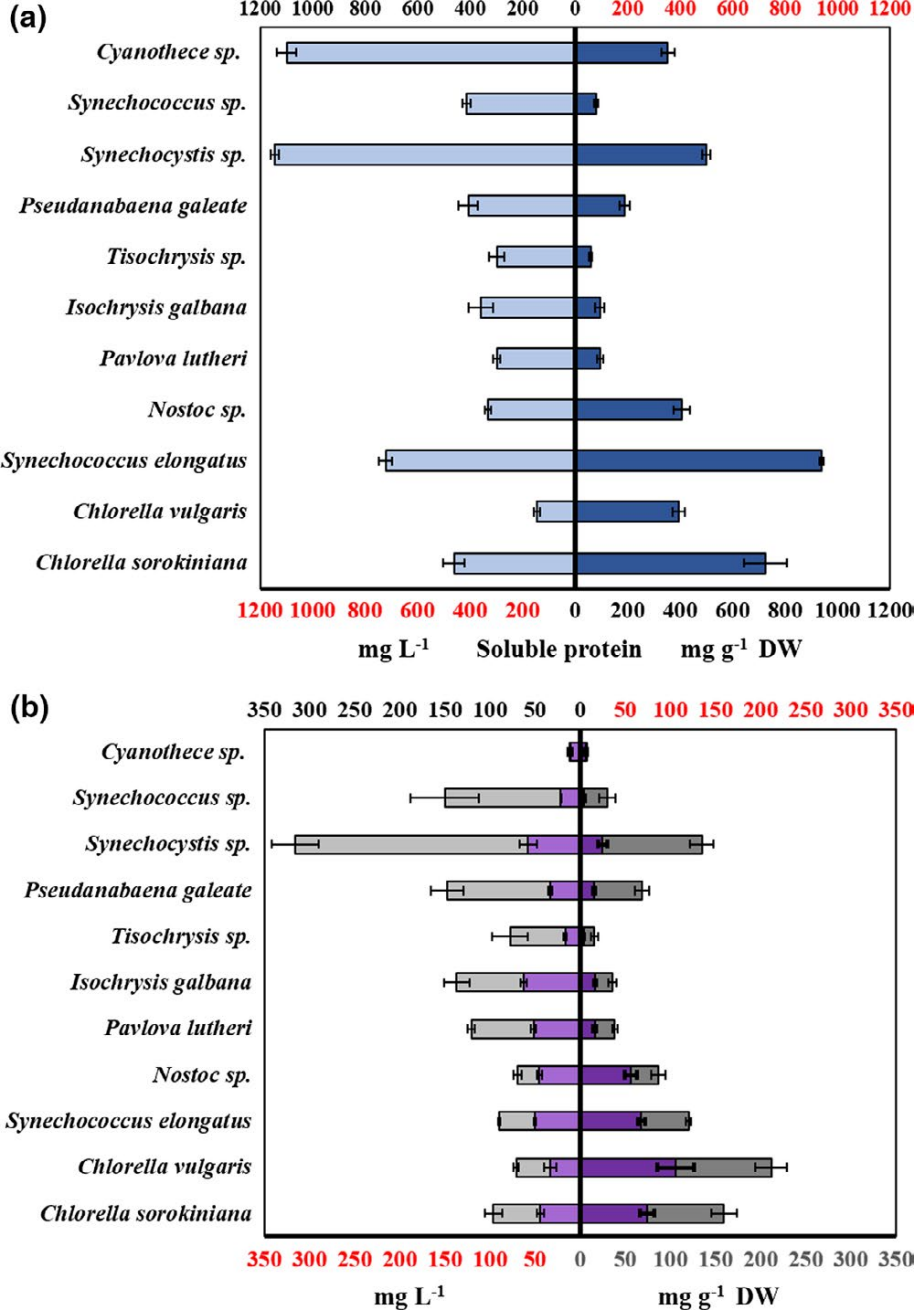
Concentrations of soluble proteins (a); (b) lipids (purple) and carbohydrates (gray) expressed in both mg L^−1^ culture medium and mg g^−1^ DW of algal biomass. (Bars show *SE* [*n* = 4])

Preliminary experiments were used to optimize cell lysis steps and to evaluate the limit of quantification (LOQ) of the macromolecule assays. It was determined that macromolecule extract was most effective with <15 mg of biomass per reaction. As the SPV lipid determination method does not require saponification before analysis, this step was removed from the protocol described by Chen and Vaidyanathan (2013), and instead, a 10‐min incubation at 70°C was included to improve protein and carbohydrate extraction. The microbiuret protein assay was a robust and sensitive method for determining protein concentrations, with the average limit of LOQ 39.08 µg ml^−1^ (*SE* = 3.75, *n = *4, *R* ^2^ = 0.998). Protein concentrations varied considerably between strains (6%–94% of total dry weight), as shown in Figure 2a. The highest protein concentration was recorded in *S*. *elongatus* UTEX 2973 (938 mg g^−1^), although in terms of protein production per liter, *Synechocystis* sp. PCC 6803 and *Cyanothece* sp. PCC 7425 ranked highest with 1.14 and 1.09 g L^−1^, respectively. The anthrone method was a similarly sensitive method, with average LOQ over four separate preliminary experiments 40.2 µg ml^−1^ (*SE *= 11.8). Carbohydrate contents varied from 12.16 to 110 mg g^−1^ (Figure 2b), with *C*. *vulgaris* and *Synechocystis* sp. PCC 6803 both containing over 10% of DW as carbohydrates. The SPV method, documented as a suitable tool for microalgal lipid analysis (Byreddy et al., 2016; Mishra et al., 2014; Park et al., 2016), was selected instead of alternatives such as gravimetric methods and the method described by Chen and Vaidyanathan (2013). In Chen and Vaidyanathan (2013), the toxic chemical chloroform and the Chemical Weapons Convention Schedule 3 substance, triethanolamine, are used to determine lipid concentrations, unsuitable for use in many laboratories. Instead, vanillin and phosphoric and sulfuric acids are used to create the color‐forming sulfo‐phospho‐vanillin (SPV) reagent. The LOQ for the SPV lipid assay was determined to be 104.8 µg ml^−1^ (*SE* = 19.7) over 4 preliminary experiments. Lipid concentrations varied from 4.12 mg g^−1^ (*Synechococcus* sp. PCC 7002/2.) to 105.8 mg g^−1^ (*C*. *vulgaris*), as shown in Figure 2b. The SPV method offered distinct advantages in terms of time, ease‐of‐use, and sample requirements compared with conventional techniques, such as gravimetric methods (Bligh & Dyer, 1959). One important consideration is the choice of standard. The SPV reagent reacts with carbon–carbon double bonds of fatty acids; therefore, the lipid profile of the selected standard should be resembling that of the microalgae (Byreddy et al., 2016). In this research, Canola oil was selected, as used by Park et al. (2016), but using oil extracted from microalgae of interest will likely lead to more accurate results. In total, the unified assay could be completed for 24 samples within 5 hours starting with a lyophilized pellet, with similar time requirements, energy, and reagent savings to the method of Chen and Vaidyanathan (2013). Although not tested in this research, the microbiuret, anthrone, and SPV assays can be performed in microplates to increase sample throughput (Cheng et al., 2011; Leyva et al., 2008).

### 3.2 Pigment identification and quantification

Microalgae biosynthesize a plethora of carotenoid molecules with significant commercial and pharmaceutical applications, including various molecules unique to microalgae. Carotenoid profiling is an important step for the discovery of strains suitable for commercialization and for monitoring how carotenoid contents change in response to changing culture conditions. Although many protocols have been devised for quantitative carotenoid analysis, interpretation is complicated by the almost identical chemical properties of different carotenoid molecules and their isomers (Rivera et al., 2011). Consequently, a multidimensional approach is necessary to identify compounds, using polarity, mass, and UV‐Vis spectrometric properties, and mass fragmentation patterns to distinguish between molecules. Algal extracts are typically resolved over C_30_ chromatography columns using liquid chromatography, with the higher pressures used in UPLC allowing greatly reduced analysis times compared with HPLC. The rapid method of Sommella et al. (2018) was slightly modified to identify carotenoid molecules from saponified ethanolic extracts from the 11 microalgal strains.

Table 1 shows the characteristic UV‐visible and mass spectra data for all peaks detected across the strains and in the authentic pigment standards. This includes 17 carotenoid peaks in the DHI‐mixed phytoplankton and individual pigment standards (bold, Table 1) and 24 additional peaks that did not match pigment standards. Of these, 11 were tentatively identified by comparison with published values (Table 1). Peak 11 was identified as all‐*trans* antheraxanthin and was a major pigment in the haptophytes (Table 2). The compound eluted from the column along with other epoxycarotenoids yet showed a spectral fine structure comparable to that of zeaxanthin (%III/II = 47%). The major mass peak was *m*/*z* 567.4 [M + H – H_2_O]^+^, although a base peak at *m*/*z* 584.5 [M]^+^ was also detected. Peaks 15 and 20 were tentatively identified as the hydroxycarotenoids nostoxanthin (*m*/*z* 601.5 [M + H]^+^) and caloxanthin (*m*/*z* 585.5 [M + H]^+^), respectively, copresent in *S*. *elongatus* UTEX 2973, *Nostoc* sp. CCNP 1411, and *P*. *galeate* CCNP 1313 and are comparable with the (*2R*,*3R*,*2′R*,*3′R*)‐nostoxanthin and (*2R*,*3R*,*3′R*)‐caloxanthin identified in both *N*. *commune* NIES‐24 (Takaichi et al., 2009) and *Synechococcus* spp. (Takaichi & Mochimaru, 2007). The myxoxanthophyll pigments, myxol 2'‐fucoside (peak 21, *m*/*z* 753.5 [M + Na]^+^) and myxol 2'‐dimethyl‐fucoside (peak 26, *m*/*z* 781.6 [M + Na]^+^), were dominant peaks in cyanobacteria (Table 2) and were easily distinguished by their characteristic UV‐visible spectra, with distinct spectral fine structures showing a bathochromic shift of ~30 nm compared with other carotenoids, as described in Takaichi et al. (2005). Interestingly, peaks 29 and 30 had identical molecular masses to that of peak 26, indicating they are isomers of Myxol 2'‐dimethyl‐fucoside or have a different sugar moiety such as a‐L‐chinovoside or a‐L‐rhamnoside (Takaichi & Mochimaru, 2007). Peak 35 was tentatively identified as 13‐ or 15 *cis*‐β‐carotene, with characteristic mass ions of *m*/*z* 536.5 [M]^+^ and 567 [M + OCH_3_]^+^ and MS^E^ fragments at *m*/*z* 444 and 430, indicating the loss of toluene [M – 92]^+^ and xylene [M +106]^+^ from the molecule, respectively. A peak at 330 in the adsorption spectra indicated a *cis*‐β‐carotene molecule. While this carotenoid was present in 7 of the strains analyzed, all‐*trans* β‐carotene was absent entirely, despite giving peaks with authentic standards. This may indicate potential isomerization of β‐carotene during the saponification step (Kopec et al., 2012) or be related to the low solubility of β‐carotene in the organic solvents used, comparatively to the other carotenoids (Craft & Soares, 1992). Peaks 1, 3, 4, and 5, present only in the haptophytes, could not be fully identified. Peak 1 had a molecular ion at *m*/*z* 599.5 and eluted at 2.45 min, much earlier than other xanthophylls with the same mass. The lack of spectral fine features (λ_max_ = 462 nm) indicates the presence of a keto group. This pigment corresponds to the “Car2” pigment detected in both *I*. *galbana* and *Tisochrysis lutea* by Serive et al. (2017). Peaks 3, 4, and 5 eluted with other compounds that masked their mass signals. These peaks showed unusual spectrophotometric properties with a hypsochromically shifted *λ*_max_ at ~400 and 430 nm. These peaks likely represent fucoxanthin derivatives. Fucoxanthin is often reported as the main carotenoid in these strains (Gilbert‐López et al., 2015); therefore, its absence is likely caused by the degradation of fucoxanthin during the saponification (Yonekura et al., 2010). Indeed, the mass spectra of Peak 1 resemble those of fucoxanthinol (*m*/*z* 599.5 [M + H – H_2_O]^+^) reported in *I*. *galbana* by Gilbert‐López et al., (2015). It is recommended that for complete, quantitative analysis of strains containing fucoxanthin or β‐carotene, or if the carotenoid profile of the strain is unknown, both saponified and saponified sample extracts are run. The other peaks could not be identified due to low concentrations of the pigments.

**TABLE 1:** Chromatographic, UV‐Vis, and mass spectroscopy characteristics of carotenoids from microalgal strains and standards

Peak	Compound	Absorbance spectra			Mass spectrometry (*m*/*z)* Mass spectrometry (*m*/*z)*				
		Rt^a^	*λ*_max_ (nm)	%III/II ^b^	Theoretical mass	Main peak	Adduct form	Low‐energy fragments	High‐energy fragments
1	Fucoxanthinol	2.45	462		616.41	599.4	M + H ‐ H_2_O^+^	581, 563	
2	**Peridinin**	3.84	457		630.36	631.5	M + H^+^	613, 553	553, 270
3	Unidentified	4.06	401, 431			509			
4	Unidentified	4.11	401, 431						
5	Unidentified	4.25	401, 431			584.5			
6	**19'‐Butanoyloxyfucoxanthin**	4.30	445, 468	28%	744.46	745.6	M + H^+^	727, 553	679, 579
7	**all‐*trans n*eoxanthin**	4.39	417, 443, 470	83%	600.42	601.5	M + H^+^		
8	fucoxanthin‐isomer	4.44	401, 431						
9	**Fucoxanthin**	4.51	450		658.42	681.5	M + Na^+^	641, 581	527, 109
10	**9'‐*cis*‐neoxanthin**	4.59	414, 437, 466	90%	600.42	601.5	M + H^+^	583, 565	565, 221, 167
11	all‐*trans a*ntheraxanthin	4.71	447, 475	47%	584.42	567.4	M + H ‐ H_2_O^+^	549, 475	549, 474, 458
12	**Prasinoxanthin**	4.81	444, 470	28%	600.42	601.5	M + H^+^	583, 565	
13	**Violaxanthin**	4.87	418, 442, 471	95%	600.42	601.5	M + H^+^		491, 221
14	**19′‐Hexanoyloxyfucoxanthin**	4.93	445, 470	45%	772.49	773.6	M + H^+^	755, 657	679, 579
15	Nostoxanthin	5.10	(427), 452, 480	29%	600.42	601.5	M + H^+^	583, 475	497, 251, 102
16	Unidentified	5.28				601.5	M + H^+^		
17	*Cis*‐*v*iolaxanthin	5.40	330, 417, 437, 466	66%	600.42	601.5	M + H^+^	583	529, 485
18	**Diadinoxanthin**	5.56	(422), 447, 477	67%	582.41	583.5	M + H^+^		
19	**Astaxanthin**	5.62	476		596.39	597.5	M + H^+^		
20	Caloxanthin	5.73	452, 479	24%	584.42	585.5	M + H^+^	615, 441	492
21	**Alloxanthin**	5.80			564.41	583.3	M + OH^+^	611, 522	
22	Myxol 2'‐fucoside	5.88	448, 475, 506	47%	730.48	753.5	M + Na^+^	567	589, 522, 187
23	**Diatoxanthin**	5.94			566.41	589.3	M + Na^+^		457
24	Unidentified	6.17	451, 470			583.5		550, 441	
25	**Lutein**	6.23	(425), 447, 475	61%	568.43	568.5	M^+^	550	469, 173
26	**all‐*trans*‐*z*eaxanthin**	6.33	453, 479	23%	568.43	569.5	M + H^+^	599, 551	550, 173
27	Myxol 2'‐dimethyl‐fucoside	6.50	450, 476, 508	53%	758.51	781.6	M + Na^+^	567	589, 215
28	Unidentified	6.73				567.5		475	
29	3’‐hydroxyechinenone	6.83	347, 450		566.41	567.5	M + H^+^	549	443, 311, 203
30	Myxol glycoside *cis*‐isomer	7.00	360, 445, 470, 500	37%		781.6	M + Na^+^	567	589, 215
31	Myxol glycoside	7.36	447, 470, 496			781.6	M + Na^+^	565, 758	589, 215
32	Unidentified	8.42	453,478	17%		567.4		536	551, 429
33	Unidentified	9.13				536.5		567	
34	Unidentified	9.26	360, (425), 447, 465			551.5			
35	Unidentified	9.33	(425), 446, 465			551.5			
36	13‐ or 15 *cis*‐β‐carotene	9.40	346, 446, 461	34%	536.44	536.5	M^+^	567	444, 430
37	**β‐carotene**	9.62	(425), 454, 479	13%	536.44	536.5	M^+^	567	444
38	**β‐cryptoxanthin**	9.66	(426), 452, 479	18%	552.43	552.5	M^+^	583	460
40	**Echinenone**	9.78	460		550.42	551.5	M + H^+^		255, 203
41	Echinenone isomer	10.0	466		550.42	551.5	M + H^+^		239, 203

^a^ Retention time on the C30 column.

^b^ Spectral fine structure calculated as the % ratio of *λ*_max_ III to *λ*_max_ II. Compounds in bold were identified by comparison with authentic standards.

**TABLE 2:** Concentrations of identified carotenoids and biomass (dry weight) in microalgal strains (mg L^−1^)

	Prasinophytes		Haptophytes			Cyanophytes					
	*Chlorella sorokiniana*	*Chlorella vulgaris*	*Pavlova lutheri* *CEFAS*1	*Isochrysis galbana* *CEFAS*2	*Tisochrysis* sp. *CEFAS*1	*Pseudanabaena galeate* CCNP 1313	*Synechocystis* sp. PCC 6803	*Synechococcus* sp. PCC 7002	*Synechococcus elongatus* UTEX L2973	*Nostoc* sp. CCNP 1411/1	*Cyanothece* sp. PCC 7725
**all‐*trans* neoxanthin**		**Trace** ^a^			**0.14** (0.04)						
**9'‐*cis*‐neoxanthin**	**0.07** (0.08)	**0.18** (0.08)	**0.29** (0.02)	**0.29** (0.15)	**0.18** (0.07)						
**all‐*trans* Antheraxanthin**		**0.73** (0.01)	**0.74** (0.05)	**0.58** (0.16)	**0.08** (0.01)						
**Violaxanthin**	**Trace**	**0.17** (0.08)	**0.30** (0.01)	**0.22** (0.10)	**Trace**						
**Nostoxanthin**						**0.06** (0.00)			**0.16** (0.02)	**0.20** (0.03)	**Trace**
**Diadinoxanthin**		**0.11** (0.00)	**0.06** (0.01)	**0.14** (0.04)	**0.19** (0.02)	**Trace**					**Trace**
**Caloxanthin**						**0.57** (0.03)			**0.37** (0.03)	**0.32** (0.04)	**0.18** (0.08)
**Myxol 2'‐fucoside**						**0.08** (0.01)	**Trace**	**0.07** (0.03)	**0.12** (0.00)	**0.12** (0.00)	**0.51** (0.03)
**Lutein**	**0.51** (0.05)	**0.32** (0.09)	**0.29** (0.00)	**0.32** (0.06)	**0.09** (0.00)	**0.09** (0.01)	**0.34** (0.28)				
**Zeaxanthin**			**0.05** (0.00)		**0.11** (0.00)	**1.87** (0.12)	**0.96** (0.17)	**1.08** (0.06)	**0.99** (0.15)	**0.21** (0.00)	**0.63** (0.03)
**Myxol 2'‐dimethyl‐fucoside**						**2.13** (1.39)	**1.98** (0.46)	**0.49** (0.33)			
**3’‐hydroxyechinenone**						**0.16** (0.02)	**0.09** (0.01)	**0.05** (0.01)	**Trace**		**Trace**
**13‐ or 15 *cis*‐β‐carotene**		**0.07** (0.00)	**Trace**	**Trace**		**0.07** (0.03)	**0.10** (0.03)	**Trace**	**Trace**	**Trace**	
**β‐cryptoxanthin**						**0.35** (0.01)	**0.30** (0.02)	**0.16** (0.01)	**0.17** (0.00)	**0.24** (0.00)	**0.20** (0.00)
**Echinenone**							**0.79** (0.16)			**0.85** (0.04)	**0.60** (0.08)
**Biomass (Dry weight)**	**625** 103	**275** 85	**3,000** **248**	**3,650** 441	**5,033** 295	**2,225** 85	**1,900** 280	**5,350** 185	**500** **58**	**725** 63	**1,850** 312

^a^ Trace carotenoids with a concentration of <0.05 mg L^−1^. Bold values denote average concentrations with standard error shown in parenthesis.

The concentrations of echinenone, β‐cryptoxanthin, lutein, and zeaxanthin (mg L^−1^ of culture medium) were calculated for all strains using calibration curves, along with the yield of biomass (mg DW L^−1^) (Table 2). Additionally, quantitative analysis was performed on 11 other tentatively identified carotenoids using an averaged molar extinction coefficient method (Table 2). Concentrations of echinenone, β‐cryptoxanthin, lutein, and zeaxanthin derived from the two calibration methods were compared using regression analysis. Coefficients between 1.1 and 1.2 and *R* ^2^ values >0.98 for these carotenoids indicated that the molar extinction method is an effective method for quantitative analysis when authentic standards are unavailable. Zeaxanthin, present in all cyanobacteria, was typically the most abundant xanthophyll, except in *P*. *galeate* CCNP 1313 and *Synechocystis* sp. PCC 6803 strains where the presiding myxoxanthophyll (either myxol 2’‐fucoside or myxol 2'‐dimethyl‐fucoside) was the most abundant. The prasinophytes and haptophytes were all characterized by the presence of 9'‐cis‐neoxanthin, violaxanthin, and lutein, while haptophytes additionally contained all‐*trans* antheraxanthin, diadinoxanthin, and *cis*‐β‐carotene (Table 2).

### 3.3 Tools for comparison of pigment profiles

**FIGURE 3:**
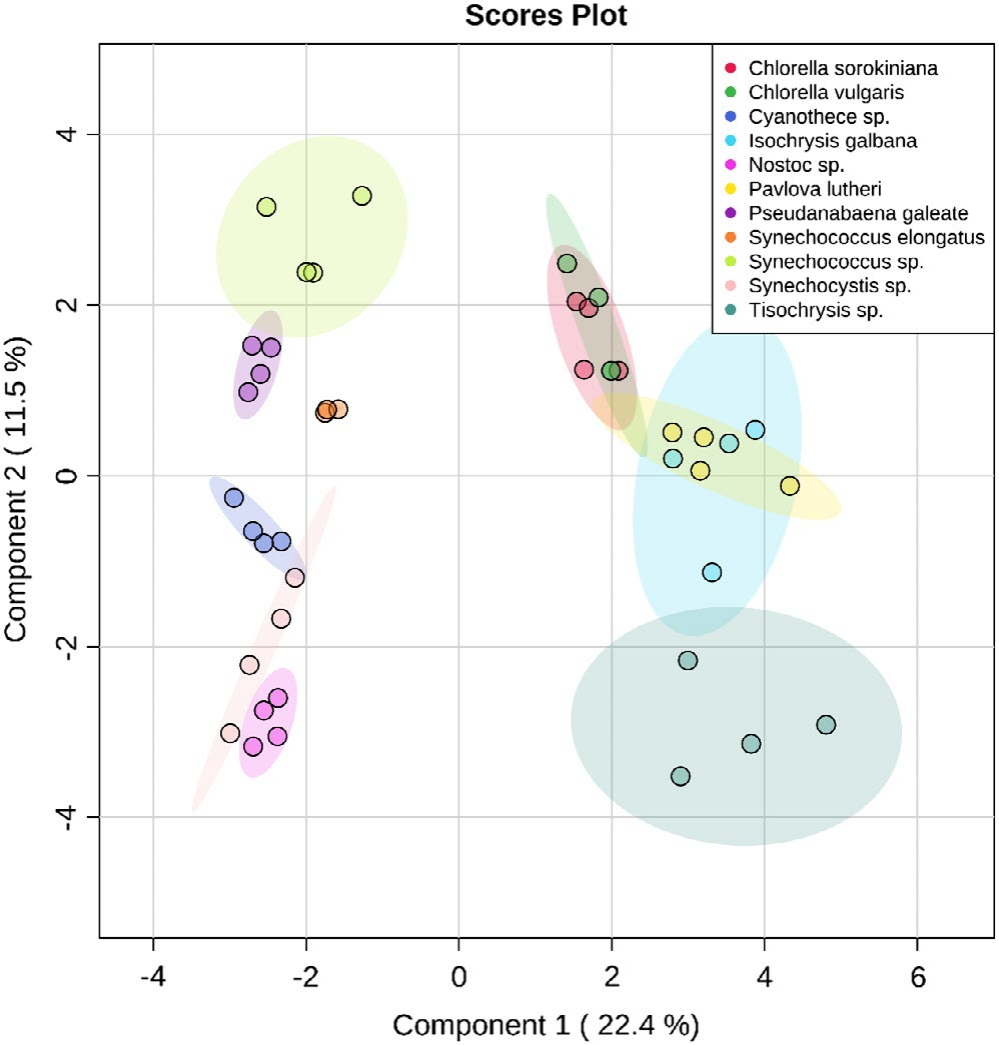
Partial least squares discriminant analysis (PLS‐DA) plot for Components 1 and 2 (a) and Components 1 and 3 (b). Shaded overalls show a 95% confidence region

Using the relative peak areas for the carotenoids identified in Table 1, PLS‐DA could distinguish between Prasinophyta, Haptophyta, and cyanobacterial strains, with genera‐level discrimination also possible for strains with Components 1 and 2 (Figure 3), and 1 and 3 (not shown). Variable importance in projection (VIP) scores indicated that 9’‐*cis*‐neoxanthin, violaxanthin, β‐cryptoxanthin, and zeaxanthin are key pigments for discriminating cyanobacteria from other phototrophic microorganisms (Component 1), whereas Components 2 and 3 were useful for resolving between genera. The data can be easily normalized, scaled, and visualized using the online MetaboAnalyst workflow or within the R environment. This approach has application both for comparing carotenoid profiles between strains and for identifying how changing growth conditions affects carotenoid biosynthesis. Alternate applications include chemotaxonomic analysis of phytoplankton in water systems and sensitive carotenoid analysis in foods and other biological samples.

In summary, the two methodologies were successfully used to determine the presence of 10 pigment and macromolecule components and generate carotenoid profiles for 11 commercially important microalgal strains. Simple, colorimetric techniques are performed in one unified assay to rapidly generate data. The method uses standard laboratory instruments and low‐toxicity reagents to determine biochemical concentrations, with wide applicability for both industrial use and research use. In the second part, a rapid, sensitive UPLC‐PDA‐MS^E^ method is outlined, which allows the identification of various commercially important and microalgae‐specific carotenoids, with methods described allowing quantification of carotenoid concentrations and simple comparison of strain pigment profiles. Forty‐one different carotenoid compounds were resolved in a 15‐min protocol, which is a distinct improvement in terms of time and resolution over many existing methods. Combining the data from both approaches, suitable strains for multiproduct refinery application can be identified, such as *Pseudanabaena galeate* CCNP 1313 for the coproduction of zeaxanthin and phycoerythrin, or *Chlorella vulgaris* for lipid, carbohydrate, and lutein production. The low sample requirements (2 ml of culture) and the high‐throughput nature of methodology also make it an ideal tool for routine culture monitoring purposes, essential for coproduct analysis in microalgal biorefineries.

## 4 CONCLUSIONS

A high‐throughput, unified method for the rapid analysis of total pigments, proteins, lipids, and carbohydrates in microalgae was developed. The assays allowed sensitive determination of commercially important components in the strains analyzed, demonstrating its applicability for rapid monitoring of macromolecules in microalgal cultures. A complimentary UPLC‐PDA‐MS^E^ protocol for high‐resolution carotenoid profiling and quantification allowed rapid identification of 41 different carotenoid compounds. The concentrations of 15 carotenoids were quantified in microalgal strains, demonstrating the applicability of the approach for carotenoid profiling and culture monitoring, with the application for optimizing pigment production and identifying candidate strains for commercial exploitation.

## CONFLICTS OF INTEREST

None declared.

## ETHICS STATEMENT

None required.

## AUTHOR CONTRIBUTION

**Joseph Palmer:** Conceptualization (lead); Data curation (lead); Formal analysis (lead); Investigation (lead); Methodology (lead); Project administration (equal); Resources (equal); Validation (lead); Visualization (lead); Writing‐original draft (lead); Writing‐review & editing (equal). **Linda Lawton:** Project administration (equal); Writing‐review & editing (equal). **Rocky Kindt:** Supervision (equal); Writing‐review & editing (equal). **Christine Edwards:** Conceptualization (equal); Project administration (equal); Supervision (equal); Writing‐review & editing (equal).

## Data Availability

The datasets used and analyzed during the current study are included in this published article.
